# 

**Published:** 1887-02

**Authors:** 


### CONTENTS.

ORIGINAL COMMUNICATIONS—

Vesical Irritation in Women. By Virgil O. Hardon, M. D., Atlanta, Ga. .... 725

Lectures on Diseases of the Skin. By Henry Wile, M. D., Atlanta, Ga. .... 737

Fluid Extracts *vs.* Tinctures. By H. S. Lott, M. D., Augusta, Ga. .... 745

Neurasthenia. By J. Edward Green, M. D., Macon, Ga. .... 750

CLINIC REPORTS—

Removal of a Giant Cell Sarcoma of the Lower Jaw. By K. C. Divine, M. D., Atlanta, Ga. .... 760

SOCIETY REPORTS—

Macon Medical Society .... 764

CORRESPONDENCE—

Our New York Letter .... 774

BOOK REVIEWS—

Transactions of the Texas State Medical Association .... 778

Books Received .... 780

EDITORIAL—

The Duties of Medical Men to Medical Societies .... 781

Items .... 759, 773, 777, 784

Our Advertisers .... 787

